# Mechanism and application prospect of ferroptosis inhibitors in improving osteoporosis

**DOI:** 10.3389/fendo.2024.1492610

**Published:** 2024-12-13

**Authors:** Jing Wang, TingRui Chen, Fei Gao

**Affiliations:** Department of Endocrinology, First Hospital of Shanxi Medical University, Taiyuan, Shanxi, China

**Keywords:** ferroptosis, osteoporosis, lipid peroxidation, iron overload, Western medicines, traditional Chinese medicines

## Abstract

Osteoporosis, a prevalent bone metabolic disorder, has emerged as a pressing global public health concern. Recent studies have illuminated a crucial link between ferroptosis and the pathogenesis of osteoporosis. Nevertheless, the intricate mechanisms underlying the role of ferroptosis in this condition remain largely unexplored. Therefore, this article comprehensively reviews the regulatory mechanisms of ferroptosis and the modulating effects on the development and progression of osteoporosis, as reported in recent years. Furthermore, this review summarizes the current state of the interventional strategies employed by both Western medicines and traditional Chinese medicines (TCMs) in addressing osteoporosis. This review aims to uncover potential novel avenues for the prevention and control of osteoporosis by synthesizing the modes of action and clinical efficacy of these therapeutic modalities.

## 1 Introduction

Apoptosis is a kind of death process of normal cells after physiological or pathological stimulation, which is active and orderly (1). The morphological characteristics of apoptosis are that the volume of cell becomes smaller, the cytoplasm is concentrated, the nuclear chromatin is condensed at the edge, the DNA is degraded, and finally multiple apoptotic bodies are formed and swallowed (2). Apoptosis exerts a crucial role in the differentiation of multicellular organisms, the development of organs, and the preservation of homeostatic balance. In recent years, as a newly discovered way of cell death, ferroptosis has been widely concerned. Ferroptosis is characterized by lipid peroxidation and iron overload, thereby promoting cell death (3), which involved in the occurrence and development of many diseases, such as tumor, Parkinson’s disease, atherosclerosis, cardiovascular disease (4–6). There are also evidences that there is a link between the progression of various liver diseases and ferroptosis, such as alcoholic liver disease (ALD), and metabolic dysfunction-associated steatohepatitis (MASH) (7, 8). Osteoporosis is a systemic skeletal disorder characterized by reduced bone mass and microarchitectural deterioration of bone tissue, leading to enhanced bone fragility and an increased propensity for fracture (9). Recent researches have uncovered an association between ferroptosis and osteoporosis, indicating that targeting the inhibition of ferroptosis may constitute a promising novel avenue for the treatment of osteoporosis. This article presents a comprehensive review of the most recent literature pertaining to the mechanisms underlying ferroptosis and pharmacological interventions, with the objective of furnishing novel research avenues for the prevention and management of osteoporosis.

## 2 Ferroptosis

### 2.1 Overview of ferroptosis

The concept of “ferroptosis” was formally proposed in 2012 by Dixon et al. (3). Ferroptosis represents a specific type of regulated cell death that is induced by iron-dependent lipid peroxidation. It is distinctly characterized by the build-up of reactive oxygen species (ROS) and the breakdown of the plasma membrane as a consequence of lipid peroxidation (10). From the perspective of morphology, ferroptosis is characterized by mitochondrial volume reduction, cell swelling, cell membrane condensation, mitochondrial ridge structure disappearance and the increase of membrane density (11). From the perspective of biochemical metabolism, it is mainly manifested as the destruction of the redox balance of cells and the increase of ROS and its derived free radicals in tissues, resulting in a large accumulation of lipid peroxides, so eventually leading to cell death (12). From the perspective of genetics, a study has found that the whole ferroptosis process is regulated by a variety of genes, and different genetic networks regulate the occurrence of ferroptosis (13).

### 2.2 Mechanism of ferroptosis

#### 2.2.1 Iron overload

Iron is an important component of many enzymes in the body, which is involved in the transport and storage of oxygen, immune regulation, DNA metabolism, inflammatory response and other processes (10). However, when the ferrous in plasma exceeds the binding capacity of transferrin, it will lead to the accumulation of a large amount of free iron, which will cause iron overload. Iron overload is an essential condition in the process of ferroptosis. A study has shown that (14) when ferrous ions are in excess, they will interact with H_2_O_2_ to undergo the Fenton reaction, which gives rise to a large accumulation of ROS and peroxidation of normal cells, ultimately leading to cell death. The reasons for iron overload may be: (1) Excessive intake of exogenous iron, such as for patients with aplastic anemia, repeated long-term blood transfusion will lead to the body’s iron levels excessive. (2) Iron metabolism related gene mutation, such as hereditary hemochromatosis (HH) is an autosomal recessive hereditary disease, which is characterized by a decrease in hepcidin activity caused by HFE gene mutation, resulting in an increase in iron into the blood (15). (3) Ineffective hematopoiesis. β-thalassemia is the most common cause of ineffective hematopoiesis. Studies have pointed out that (16, 17) the cause of iron overload in β-thalassemia is mainly caused by the increase of ineffective erythropoiesis, which leads to the continuous high expression of ferroportin (FPN) on the surface of small intestinal villus cells, so the absorption of iron is increased. Furthermore, menopause, obesity and excessive ingestion of vitamin C have also been documented to augment the susceptibility to iron overload (18–20).

#### 2.2.2 Lipid peroxidation

Lipid peroxidation is related to the process of lipid peroxidation, which is mediated by free radicals and occurs on cell membranes and organelle membranes. Lipids constitute the main components of cell membranes and play a key role in maintaining cell structure and physiological functions. The cell membrane contains a large amount of polyunsaturated fatty acids (PUFAs), which are easily oxidized due to their special structure and are the main targets of ROS attacks, especially arachidonic acid (AA) and adrenergic acid (AdA) (21). The process of lipid peroxidation includes three steps, the first free AA or AdA is activated by acyl-CoA synthetase long-chain family member 4 (ACSL4) to form AA-CoA or AdA-CoA (22). Subsequently, AA-CoA or AdA-CoA was catalyzed by lysophosphatidylcholine acyltransferase 3 (LPCAT3) to synthesize AA-PE or AdA-PE with phosphatidylethanolamine (PE) in membrane phospholipids (23). Finally, lipid peroxidation occurred in AA-PE or AdA-PE. Lipid peroxidation can be mediated via two pathways (24). One is the ROS non-enzymatic pathway. When the ferrous ion concentration in the cell is excessive, the occurrence of the Fenton reaction will lead to a significant accumulation of ROS. ROS reacts with PUFAs in the lipid membrane and induces lipid peroxidation to form lipid peroxides, such as malonaldehyde (MDA). High concentrations of lipid peroxides can cause oxidative stress in cells, which is a necessary condition for the initiation of ferroptosis (25). The other is lipoxygenases (LOXs) enzymatic pathway, LOXs are iron-containing proteins that directly oxidize PUFAs on the biofilm to participate in ferroptosis, causing rapid and severe damage to the cell membrane (26). Recent studies have shown that ACSL4 mutations are associated with the occurrence and development of many diseases, such as tumors, cardiovascular diseases, and bone diseases (27–29). ACSL4 is a key protein in the process of ferroptosis, which can induce lipid peroxidation of polyunsaturated fatty acids and promote ferroptosis. Dolma S et al. found that (30) knocking out the ACSL4 gene can effectively reduce glutathione peroxidase 4 inhibitor-induced ferroptosis, which may be a new idea to inhibit ferroptosis.

#### 2.2.3 ROS accumulation

Oxidative stress is a pathological state of the imbalance between peroxidation and antioxidant system in the body. Under normal circumstances, even minor fluctuations in the redox balance within the biological system can be promptly sensed by a specialized “sensor”. After that, the downstream signal transduction cascade is activated to regulate the metabolic process. However, when the intracellular H_2_O_2_ exceeds a certain threshold, the integrity of the cell begins to be affected (31). JIA (32) and HE (33) et al. found that intracellular ROS production was significantly increased in a high-iron environment and increased in a dose-dependent manner with iron. When a large amount of iron is accumulated in the body, the ferrous in the cells will increase, because of the instability and high reactivity of ferrous, Fenton reaction occurs between ferrous and H_2_O_2_, resulting in the production and aggregation of hydroxyl (OH) (14). The imbalance in the rate of ROS production can lead to oxidative stress, which in turn produces free radicals, which can cause damage to cellular DNA, proteins, and enzyme activity, and ultimately cause cell death (34). Glutathione (GSH), which is constituted by glutamic acid, cysteine, and glycine, serves as a crucial antioxidant and free radical scavenger within cells. Glutathione peroxidase 4 (GPX4), being the fourth member of the selenium-containing GPX family, is regarded as an enzyme isomer that is closely associated with ferroptosis and functions as a core regulator of ferroptosis. The primary reason for this is that it is the sole enzyme in the cell capable of reducing lipid peroxides (35). It can convert lipid peroxides into lipid alcohols, thereby impeding the generation of toxic ROS (36). Nevertheless, when GSH is depleted, the activity of GPX4 is lost and the antioxidant capacity of cells is diminished, which will accelerate the elevation of lipid peroxidation within cells and give rise to ferroptosis (37).

### 2.3 Regulation of ferroptosis

#### 2.3.1 Regulation of ferroportin

As an essential trace element for the human body, iron ions are involved in a variety of physiological activities and metabolic processes in cells. Iron in food is first absorbed by epithelial cells in the small intestine and converted into ferrous, which is then transferred into the cytoplasm of intestinal epithelial cells by divalent metal ion transporter (DMTI) to release. The released ferrous enters the labile iron pool (LIP) or is stored in ferritin in a stable form of iron ions to meet the physiological metabolic function of cells. FPN, as the only membrane transporter that mediates iron ion export, can release excess iron into the blood and eventually be utilized in various tissues and organs. It is expressed on the cell membranes of liver, duodenal epithelial cells and reticuloendothelial macrophages (38). When the body accumulates a large amount of iron due to various factors, the excess ferrous ions will interact with H_2_O_2_ and cause Fenton reaction to cause death. And when FPN is mutated, it will also cause abnormal iron metabolism and lead to ferroptosis (39). Hepcidin is a hormone-like polypeptide synthesized and secreted by liver cells. Nemeth et al. (40) found that in the process of iron metabolism regulation, the combination of FPN and hepcidin can lead to the degradation of FPN, so that iron cannot be discharged into the blood circulation, and reducing the iron level in the blood. Nemeth et al. (40) found that the combination of FPN and hepcidin can reduce the level of iron in the blood during the regulation of iron metabolism. This is because the binding of hepcidin to FPN1 can accelerate the ubiquitination, internalization and degradation of FPN1, and reduce the serum iron concentration by reducing the absorption of iron by intestinal epithelial cells and reducing the release of iron by hepatocytes and macrophages. Therefore, regulating iron homeostasis through hepcidin may be a new target for the treatment of ferroptosis. However, when the level of hepcidin increases significantly, the concentration of iron ions in plasma decreases, resulting in iron not being fully used for the synthesis of hemoglobin, resulting in anemia (41). At the same time, in the state of chronic inflammation, the expression of hepcidin may increase, which inhibits the absorption and utilization of iron and further aggravates anemia (42). This may be a side effect of hepcidin. Therefore, it is necessary to strictly control the intake of hepcidin-like drugs to maintain normal iron homeostasis and prevent anemia caused by iron deficiency and tissue damage and organ failure caused by excessive iron.

#### 2.3.2 Regulation of enzyme

##### 2.3.2.1 GSH/GPX4/System Xc- pathway

The emergence of ferroptosis is predominantly attributed to the inactivation of the cellular antioxidant system, subsequently leading to the build-up of lipid peroxides. GSH/GPX4/System Xc-pathway plays a leading role in this process (43). System Xc- is a transmembrane protein composed of heterodimers of solute carrier family 7 member 11 (SLC7A11) and recombinant solute carrier family 3 member 2 (SLC3A2), which is related to the synthesis of GSH. System Xc- can exchange intracellular and extracellular cystine with glutamate at a ratio of 1:1 to maintain normal cell metabolism (44). System Xc- can work with GPX4 to exert System Xc- antioxidant effect, when it is inhibited, intracellular GSH synthesis is reduced, which in turn induces ferroptosis. A study have shown that (45) the ferroptosis inducer erastin induces ferroptosis by inhibiting System Xc-, which indirectly affects the activity of GPX4 and eventually leads to ferroptosis. At the same time, a study has pointed out that (46) the transcription factor involved in the oxidative response (ATF3) can regulate the expression of GPX4 by inhibiting SLC7A11 in high glucose environment, and eventually leads to ferroptosis. In addition, the p53 gene is known to be a tumor suppressor gene that regulates cell growth by promoting apoptosis and tissue repair. There is a study that shows that the combination of P53 and nuclear factor E2-related factor 2 (Nrf2) can also reduce the uptake of cystine by System Xc-, indirectly inhibit the activity of GSH and GPX4, and then cause the accumulation of ROS, eventually leads to ferroptosis (47).

##### 2.3.2.2 NADPH/FSP1/coenzyme Q pathway

Ferroptosis-suppressor-protein 1 (FSP1) is a flavin protein, which is also known as apoptosis-inducing factor mitochondria-associated 2 (AIFM2) because of its structural similarity to apoptosis-inducing factor (48). Recent research indicates that FSP1 is not only involved in apoptosis, but also plays an important role in ferroptosis (49), this is mainly because its domain includes reduced nicotinamide adenine dinucleotide (NADH) oxidoreductase, which has NAD (P) H oxidase activity and can reduce ubiquinone 10 (CoQ 10) to dihydroubiquinone (CoQH2). CoQH2 can scavenge ROS and lipid free radicals in cells, thereby inhibiting ferroptosis (50). DOLL et al. (49) found that the function of FSP1 in inhibiting ferroptosis is independent of the GSH/GPX4/System Xc- pathway. However, there is also evidence that (51), in the case of normal GPX4 expression levels, the absence of FSP1 will also lead to an increase in peroxides, indicating that this pathway and the GSH/GPX4/System Xc- pathway also have a synergistic effect. Therefore, inhibiting ferroptosis by regulating FSP1 activity provides an innovative paradigm for drug exploration and therapeutic intervention of diseases.

##### 2.3.2.3 GTP/GCH1/BH4 pathway

GTP/GCH1/BH4 pathway is another way to regulate ferroptosis independent of GSH/GPX4/System Xc^-^. BH4 plays an important role in angiogenesis, inflammation, oxidative stress, etc. However, BH4 has a direct antioxidant effect to protect cells from ferroptosis, and can also inhibit ferroptosis by *de novo* synthesis of COQ10 in this pathway (52). GTP cyclohydrolase-1(GCH1) is the rate-limiting enzyme in BH4 synthesis and plays a role in regulating BH4 content, GCH1 plays a role mainly through its metabolites tetrahydrobiopterin and dihydrobiopterin (53). Kraft et al. discovered (52) that the overexpression of GCH1 not only eradicates lipid peroxidation but also substantially suppresses ferroptosis. Moreover, the protective effect of GCH1 on cells is independent of the proteins associated with the known iron metabolism pathway or the glutathione system. Therefore, the GTP/GCH1/BH4 pathway, as an endogenous antioxidant pathway, inhibits the occurrence of ferroptosis through a mechanism independent of the GSH/GPX4/System Xc- pathway, which may provide a new target for the prevention and treatment of ferroptosis.

#### 2.3.3 Ferritinophagy

**Figure 1:**
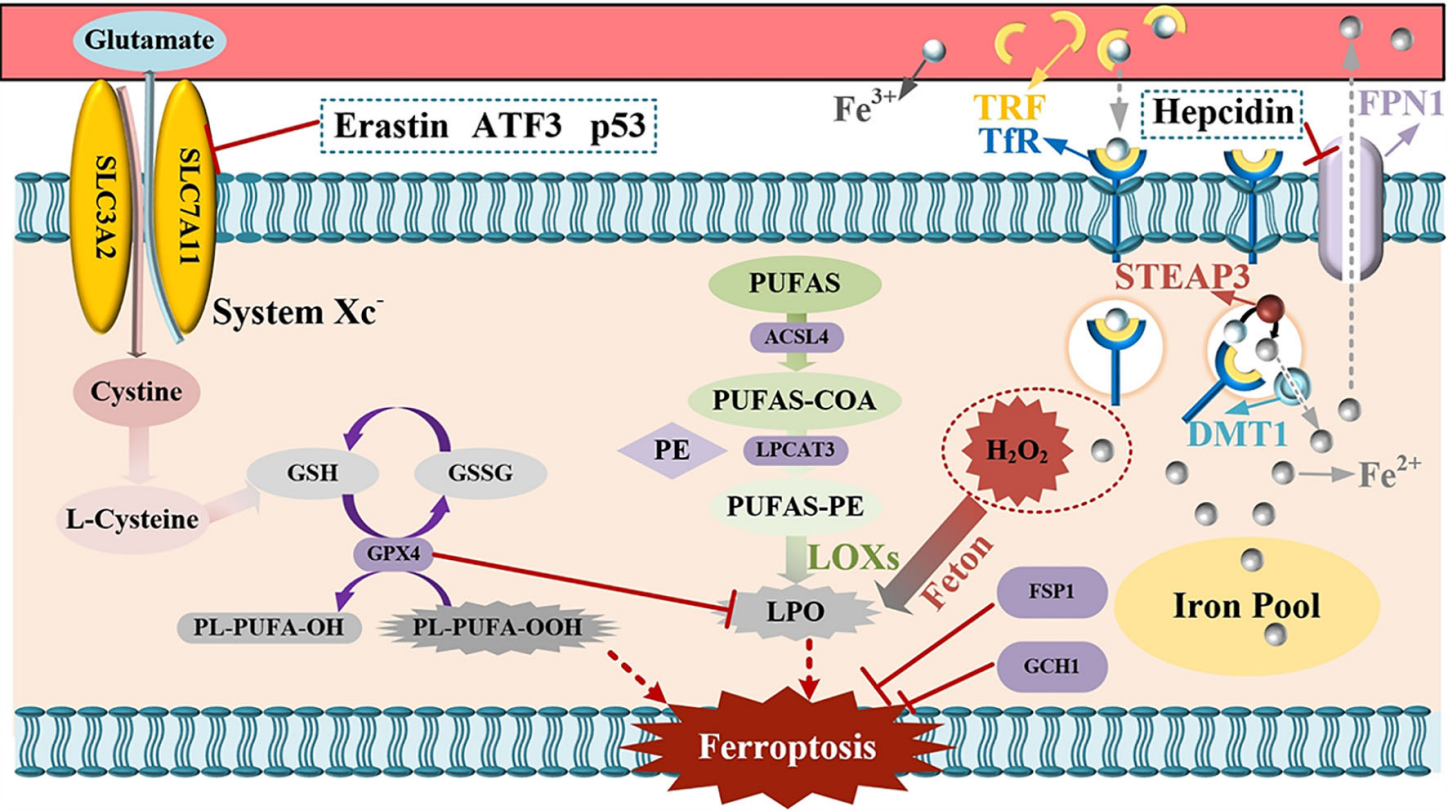
Mechanism and regulation of ferroptosis.

Ferritinophagy, which is mediated by nuclear receptor coactivator 4 (NCOA4), is an important part of the ferroptosis pathway. It can specifically recognize ferritin heavy chain (FTH) and transport it to lysosomes to complete autophagy (13). Ferritinophagy is the only way for intracellular ferritin to be converted into available iron ions. In the process of ferritinophagy, the ferritin protein itself is not converted to iron ions. Rather, the protein is degraded and recycled and the iron ions that were stored are released. However, under certain pathological conditions, ferritinophagy can be over-activated and cause cell damage. Li et al. found that (54) that deferoxamine can inhibit the expression of NCOA4 in cardiomyocytes of diabetic rats, thereby inhibiting ferritinophagy. At the same time, studies have shown that (55, 56) hypoxia inducible factor-1α (HIF-1α) and EC-Exos from endothelial cells can also inhibit ferroptosis by inhibiting ferritinophagy. In addition, mitophagy can remove damaged mitochondria in cells to maintain cell homeostasis. In recent years, a study has pointed out that it can cause iron and ROS accumulation, and induce ferroptosis (57). The specific mechanism is shown in **Figure 1**.

TRF, denoting transferrin, and TfR, signifying transferrin receptor, upon their combination, facilitate the translocation of iron ions present in the bloodstream into cells. The iron ions that have entered the cells are converted into ferrous ions under the catalytic action of STEAP3. DMT1, namely divalent metal transporter 1, is capable of discharging ferrous ions into the cytoplasmic compartment. The released ferrous ions then enter the labile iron pool. In the event of an excessive iron ion concentration, the ferrous ions will interact with H_2_O_2_, thereby triggering the Fenton reaction and culminating in ferroptosis. FPN1, which refers to Ferroportin, is responsible for releasing surplus iron into the blood. Hepcidin, on the other hand, can suppress the function of FPN and consequently reduce the iron levels in the blood. PUFAs, or polyunsaturated fatty acids, are ultimately converted into lipid peroxides under the influence of diverse enzymes, giving rise to oxidative stress and subsequent cell death. GPX4, that is glutathione peroxidase 4, in conjunction with System Xc-, undertakes an antioxidant role. When System Xc- is inhibited, the synthesis of intracellular glutathione (GSH) is diminished, leading to an augmentation in peroxides and consequent cell death. Erastin, ATF3, and P53 are capable of inhibiting System Xc- and thereby inducing ferroptosis. FSP1, or ferroptosis-suppressor-protein 1, and GCH1, which is GTP cyclohydrolase-1, possess the property of inhibiting ferroptosis.

## 3 Ferroptosis and osteoporosis

### 3.1 Ferroptosis and BMSCs

Bone marrow mesenchymal stem cells (BMSC) are tissue stem cells with multi-directional differentiation potency, which can differentiate into a variety of cell lineages, including bone cells, adipocytes and cardiomyocytes. This unique property enables BMSCs to play a crucial role in therapeutic interventions for many diseases, as they can potentially promote tissue repair and regeneration through their ability to differentiate. Under the influence of various physical and chemical factors or external stimuli, the transformation of BMSCs into osteoblasts is impaired, and the number of osteoblasts is decreased. Therefore, the balance of bone homeostasis is broken, eventually leading to ferroptosis. In recent years, with the study of the field of ferroptosis, many scholars have found that there is a close relationship between ferroptosis and BMSCs. The Wnt/β-catenin signaling pathway has been known to promote osteogenic differentiation (58). A number of studies have pointed out that (59–61), iron overload induces ferroptosis by inhibiting the Wnt/β-catenin signaling pathway of BMSCs, down-regulating the expression of osteogenic differentiation-related genes, such as Runt-related transcription factor 2 (RUNX2) and inducing the production of ROS. Li et al. (62) found that high-dose dexamethasone can induce the ferroptosis of BMSCs by blocking the activation of PI3K/AKT signaling pathway to inhibit the osteogenic differentiation of BMSCs. The PI3K/AKT signaling pathway mainly can stimulate the differentiation and growth of osteoblasts and inhibit their apoptosis (63). In addition, Zhang et al. (64) found that high glucose could increase ROS and lipid peroxidation products in BMSCs, thus causing ferroptosis in BMSCs, and when ferroptosis inhibitors were used, the osteogenic differentiation and proliferation of BMSCs could be restored.

### 3.2 Ferroptosis and osteoblasts

Studies have found that (64, 65) iron overload can inhibit the activity of osteoblasts, thereby affecting their differentiation and mineralization process, and eventually lead to reduce bone mass or even fracture. The main factor leading to bone loss is that when intracellular ferrous ions are overloaded, the Fenton reaction is initiated, which leads to osteoblast dysfunction. Tian et al. (66) found that ROS induced by iron overload cause osteoblast necrosis by stimulating receptor-interacting protein kinase-1 (RIPK1) phosphorylation, when treated with ROS inhibitors, the activity of osteoblasts was significantly improved and the necrosis rate of osteoblasts was reduced. Nrf2/HO-1 signaling pathway also plays an important role in regulating oxidative stress. A study has shown that (67) high glucose inhibits osteogenesis mainly by inhibiting the Nrf2/HO-1 signaling pathway to promote iron overload and lipid peroxidation in osteoblasts of T2DM.

In addition, one study indicates that (68) iron overload in high glucose environment is caused by the overexpression of DMTI in osteoblasts. However, Poly (rC) -binding protein 1 (PCBP1) can effectively protect osteoblasts by increasing the expression of ferritin in cells and reducing the occurrence of ferroptosis in high glucose environment (69). It has been reported that HIF-1α can mediate the adaptability of the body to oxidative stress under hypoxic conditions by regulating ROS (70). In addition, HIF-1α can also regulate GPX4 by promoting the synthesis of GSH, which plays an indirect role in inhibiting ferroptosis of osteoblasts (71). Mitochondrial ferritin (FtMt) is a protein that stores ferrous ions in mitochondria. Wang et al. (72) found that in diabetic osteoporosis, FtMt can reduce the content of ROS in osteoblasts, thereby reducing the occurrence of ferroptosis.

### 3.3 Ferroptosis and osteoclasts

Osteoclasts are derived from mononuclear phagocyte system of hematopoietic stem cells. The key step of osteoclast differentiation is that (73) receptor activator of nuclear factor-κB ligand (RANKL) produced by osteoblasts binds to receptor activator of nuclear factor-κB (RANK) on osteoclast precursor cells, thereby activating NF-κB signaling pathway and promoting osteoclast differentiation. Ni et al. found that (55) when RANKL was stimulated, the level of GSH in osteoclasts decreased, and the expression of MDA and prostaglandin-endoperoxide synthase 2 (PTGS2) increased. It is worth mentioning that RANKL failed to induce ferroptosis under hypoxia, which may be due to the effect of oxygen on the ferroptosis process of osteoclasts. At the same time, a study has shown that (74) iron chelating agents such as deferoxamine (DFO) can inhibit osteoclast formation *in vitro*, which further confirms that ferroptosis is involved in osteoclast formation. Recent research indicates that (75) zoledronic acid can induce ferroptosis in osteoclasts, which is mainly achieved by FBXO9-mediated p53 ubiquitination and degradation. Nrf2 is a key transcription factor regulating cellular antioxidant response. Zhang et al. found that (76) Nrf2 has the ability to enhance antioxidant capacity and mediate the up-regulation of ferroportin 1 (FPN1) and ferritin leading to a decrease in iron levels in osteoclasts, which is essential for the differentiation of bone cells. Artemisinin is an antimalarial drug isolated from Artemisia annua. In recent years, more and more studies have reported that artemisinin and its derivatives can be used as potential alternative medicines for the treatment of bone loss. The mechanism may be that (77) artemisinin and its derivatives inhibit osteoclast differentiation by down-regulating RANKL-induced osteoclast production, leading to ferroptosis of osteoclasts.

Iron overload regulates the activities of osteoblasts and osteoclasts through multiple pathways. In terms of oxidative stress, iron overload leads to an increase in ROS within osteoblasts, which causes damage to the cell membrane and activates the Nrf2/HO-1 signaling pathway, thereby impeding bone formation (67). For osteoclasts, the activation of the NF - κB pathway by ROS results in an increase in the expression of key enzyme genes, thus enhancing bone resorption (73). In the iron regulatory protein pathway, under normal conditions, iron regulatory proteins (IRP) can bind to iron-responsive elements (IRE) to regulate the expression of genes related to iron metabolism. In osteoblasts, iron overload induces an imbalance in the binding of IRP1 and IRE, leading to an increase in ferritin synthesis and subsequent toxicity. Abnormal IRPs in osteoclasts affect iron metabolism, disrupt the mitochondrial respiratory chain, and generate more ROS, thereby enhancing their activity (78). In the regulation of cytokines, iron overload prompts osteoblasts to reduce the secretion of bone formation factors and increase the secretion of inhibitory factors. Osteoclasts are activated due to an increase in the ratio of RANKL to osteoprotegerin (OPG) caused by iron overload (79).

However, so far, there are still few studies on the correlation between ferroptosis, BMSCs, osteoblasts, osteoclasts and OP, and the mechanism still needs to be further explored.

## 4 Application of ferroptosis inhibitor in the treatment of osteoporosis

### 4.1 Western medicines

#### 4.1.1 Iron chelator

Deferoxamine (DFO), deferiprone (DFP) and deferasirox (DFS) are the three iron chelators currently used in clinical practice, among them, DFO is the longest and most widely used iron chelator (80). A Study has confirmed that (81) DFO can improve iron overload and restore bone-related indicators. DFO is a HIF-1α activator with dual protective effects. It not only inhibits ferroptosis in osteoblasts, but also protects osteoclasts from ferroptosis by reducing RANKL-induced ferritin autophagy. Therefore, DFO can reduce ferroptosis by reversing elevated iron levels in OP patients. There is also evidence that (82) DFO can promote the differentiation of BMSCs into osteoblasts through the Wnt/β-catenin signaling pathway. Ding et al (83) confirmed that DFO can activate the PI3K/AKT signaling pathway. The activation of the PI3K/AKT signaling pathway is also considered to directly promote the osteogenic differentiation of stem cells, so, it can be speculated that the promotion of DFO osteogenesis may be related to the activation of the PI3K/AKT signaling pathway (84). DFP is the first clinically available oral iron chelator. Link et al. found that (85) DFP can reduce oxidative stress damage caused by iron overload. Naves et al. found that (86) DFP can reduce the concentration of iron ions in osteoblasts and increase the activity of alkaline phosphatase (ALP), which is stronger than DFO. DFS is mainly used in patients over 2 years of age with chronic iron overload in children with thalassemia and iron overload caused by other transfusion-dependent diseases. A study has shown that (87) DFS can inhibit osteoclast ferroptosis by inhibiting the activity of NF-κB pathway, while DFO and DFP have no such effect. Therefore, it is of great significance to use different iron chelators for the prevention and treatment of osteoporosis.

#### 4.1.2 Melatonin

Melatonin (MT), N-acetyl-5-methoxytryptamine, was originally considered to be a hormone that regulates biological rhythms. In recent years, a study has found that (86) MT not only has the effect of regulating sleep, but also has anti-inflammatory, anti-oxidation, anti-osteoporosis, anti-tumor and other effects. The main mechanisms of the effect of MT on bone metabolism are as follows: (1) Promote the proliferation and differentiation of osteoblasts. MT can promote the proliferation and differentiation of osteoblasts through direct or indirect ways, thereby delaying the process of OP. Maria et al (88) found that MT could increase the expression of RUNX2 and inhibit the expression of peroxisome proliferators-activated receptors (PPARγ) and RANKL by adding MT to the co-culture model of BMSCs and monocytes, which indicated that MT could promote the differentiation of BMSCs into osteoblasts. Chen et al. found that (89) MT can reduce the level of oxidative stress in BMSCs of osteoporotic rats and indirectly promote osteoblast differentiation, thereby improving OP symptoms. Guan et al. found that (90) MT can weaken the osteogenic inhibition of BMSCs referred to by lipopolysaccharide and indirectly promote osteogenic differentiation, and this effect may be achieved through the TLR4/NF-κB signaling pathway. At the same time, it has been found that (91) MT can significantly promote the expression of bone morphogenetic protein (BMP), thereby promoting the proliferation and differentiation of osteoblasts and increasing bone mass. In addition, a study has shown that (92) MT can promote bone formation by increasing the expression of osteocalcin (OCN), thereby promoting the proliferation and migration of MSCs and enhancing the proliferation and differentiation of osteoblasts, and ultimately increase bone mass.(2) Inhibit osteoclast differentiation formation. Zhou et al. (93) found that MT can reduce intracellular ROS and inhibit osteoclast formation, thereby reducing bone resorption and increasing bone mass. In addition, they also speculated that MT may inhibit osteoclast differentiation by regulating NF-κB signaling pathway. Under the condition of high glucose, the level of iron autophagy in osteoblasts increases, which promotes the occurrence of ferroptosis. One study indicates that (94) MT can inhibit ferroptosis of osteoblasts under high glucose conditions, the mechanism is not clear, which may be through up-regulating the expression of miR-550a-3p. MiR-550a-3p is correlated with changes in bone tissue morphological parameters and microstructural parameters, which may provide a reference for bone quality assessment and fracture risk prediction of OP.

#### 4.1.3 Ferrostatin-1

Ferrostatin-1 (Fer-1) is an aromatic amine antioxidant and an effective ferroptosis inhibitor. It can specifically prevent the accumulation of ROS produced by lipid peroxidation. Wu et al (95) induced ferroptosis in mouse renal tubular epithelial cells by high glucose, and found that the content of ferrous and MDA in the kidney tissue of mice in the Fer-1 intervention group decreased, and the GSH content increased significantly. This indicates that Fer-1 can increase the concentration of GSH in diabetic kidney tissue, inhibit lipid oxidation and iron overload, thereby improving ferroptosis. In addition, they also found that the protein expression levels of GPX4 and SLC7A11 in this group of mice were up-regulated, which further indicated that Fer-1 inhibited ferroptosis by up-regulating the activity of GPX4 and SLC7A11. Jiang et al. (96) found that iron overload destroyed the redox balance of cells by establishing an iron overload mouse model, which was mainly manifested by excessive intracellular ROS, lipid peroxidation, increased MDA, and decreased superoxide dismutase (SOD) and GSH. In addition, they also found that the levels of OCN, OPN and P1NP in this group of mice serum were decreased, while the use of Fer-1 could improve the above-mentioned peroxidation state and significantly increase the level of OPN. Zhang et al. discovered that (64) high glucose suppresses the osteogenic differentiation and cell proliferation activity of BMSCs, leading to the accumulation of ROS and lipid peroxides within cells. However, following the addition of Fer-1, the levels of ROS and lipid peroxides declined, implying that the inhibitory effect of high glucose on the osteogenic differentiation of BMSCs might be induced by ferroptosis. So, Fer-1 may provide new ideas for the prevention and treatment of osteoporosis in the future.

#### 4.1.4 Other Western medicines

Vitamin D is essential for bone mineralization and bone quality maintenance. Among them, 1,25 (OH) D is the most active metabolite of vitamin D, which plays an anti-osteoporosis role by binding to vitamin D receptor (VDR) (97). Xu et al. (98) found that VDR activation can decrease osteoblast ferroptosis by stimulating Nrf2/GPX4 signaling pathway. Zileuton is a N-acetyl-5-methoxytryptamine that inhibits the production of ROS in the cytoplasm and has a significant protective effect on erastin-induced ferroptosis (99). In addition, Maresin 1 is a polyunsaturated fatty acid necessary for human body. Zhang et al. found that (100) Maresin 1 can protect osteoblasts from ferroptosis in high glucose environment by affecting Nrf2 signaling pathway, thereby improving osteogenic ability. A summary of the various Western medicines is found in **Table 1**.

**Table 1:** Summary of various Western medicines.

Western medicines	Regulatory pathway	Operation
Deferoxamine	Wnt/β-catenin PI3K/AKT	Inhibit ferroptosis of osteoblasts and osteoclasts
Deferiprone	—	Reduce the concentration of iron ions in osteoblasts Reduce oxidative stress injury
Deferasirox	NF-κB	Inhibition of osteoclast ferroptosis
Melatonin	TLR4/NF-κB	Reduce intracellular reactive oxygen species levels Increase GPX4 activity Inhibit ferroptosis induced by high glucose
Ferrostatin-1	—	Improved peroxidation Increased OPN levels
Vitamin D	Nrf2/GPX4	Reduce osteoblast ferroptosis
Zileuton	—	Inhibition of peroxide production
Maresin 1	Nrf2	Inhibit ferroptosis induced by high glucose

### 4.2 Traditional Chinese medicines

#### 4.2.1 Icariin

Icariin is a flavonoid compound in epimedium, which is widely used in bone metabolic diseases and has pharmacological effects such as anti-osteoporosis and anti-tumor (101). Li found that (102) the level of MDA in BMSCs added with icariin decreased and the level of SOD increased, which indicated that icariin can inhibit the accumulation of ROS in BMSCs caused by Erastin to reduce apoptosis. Fu et al. (103) found that icariin can delay the inhibition of cystine metabolism by down-regulating FPN1 and up-regulating GPX4, inhibit lipid peroxidation and iron metabolism, and ultimately reduce ROS to inhibit osteoblast death. In addition, they also observed that icariin can regulate osteoblast proliferation and differentiation by increasing osteoblast Runx-2 and ALP protein expression. In addition, a study has shown that (104) icariin can inhibit mitochondrial oxidative stress and ferroptosis by targeting Nrf2. Therefore, icariin can prevent bone loss caused by iron overload and thus play a role in the prevention and treatment of osteoporosis.

#### 4.2.2 Baicalein

Baicalein is a monomeric compound mainly derived from the root of Scutellaria baicalensis Georgi. It has antioxidant, anti-inflammatory, anti-tumor and anti-allergic properties (105). Xie et al. found that (106) baicalein is a natural ferroptosis inhibitor, which can inhibit erastin-induced GPX4 degradation, thereby protecting cells from membrane lipid peroxidation. One study indicates that (107) the mechanism of baicalein inhibiting ferroptosis and reducing bone damage may be related to the up-regulation of SLC7A11/GPX4 pathway and the promotion of BMSCs proliferation and osteogenic differentiation. Guo found that (108) baicalein at an appropriate concentration can increase the expression of GPX4 and β-catenin protein in ferroptosis model cells, increase the activity of cell proliferation, and then inhibit ferroptosis. This reveals that baicalein may up-regulate GPX4 through the Wnt/β-catenin signaling pathway to inhibit ferroptosis.

#### 4.2.3 Quercetin

Quercetin is a natural flavonoid compound that is widely found in many Chinese herbal medicines. Quercetin and its derivatives have anti-inflammatory, antioxidant, antibacterial, and anti-tumor effects (109), among them, quercetin has obvious advantages in anti-oxidation and anti-inflammatory. A Study has pointed out that (110), quercetin mainly enhances its antioxidant properties by regulating GSH levels, regulating enzymes or antioxidants. Yin et al.found that (111) quercetin can reduce the expression of ATF3, restore the activity of GPX4, and reduce ROS, thereby reducing the inflammatory response. At the same time, recent research indicates that (112) quercetin can improve lipopolysaccharide-induced osteoblast apoptosis, which may be achieved by regulating MAPK and Wnt/β-catenin pathways, thereby promoting osteoblast proliferation, differentiation and mineralization.

#### 4.2.4 Myricitrin

Myricitrin is a potent antioxidant isolated from plants, which has analgesic, anti-inflammatory, neuroprotective and antioxidant effects (113). A study has found that (114), myricitrin can reduce ROS production, aldehyde levels and increase GSH activity, thereby reducing the inflammatory response, and it can improve the microstructure of bone. Obviously, the therapeutic effect of myricetin on osteoporosis is related to the regulation of oxidative stress-mediated ferroptosis. Wang found (115) that myricitrin can inhibit RANKL-induced osteoclast precursor cells to osteoclast differentiation and maturation, and the degree of inhibition is proportional to the concentration of myricitrin. Therefore, inhibiting the formation and activity of osteoclasts is of great significance for the development of new drugs for the treatment of OP.

#### 4.2.5 Other traditional Chinese medicines

A study have shown that (116) low concentration of benzoylaconitine can increase the expression of GPX4 and β-catenin protein in osteoblasts induced by Erastin, and effectively inhibit the occurrence of ferroptosis. Duzhong-duanduan, luteolin and echinacoside can significantly inhibit the expression of p53 protein, reduce osteoblast apoptosis, promote osteogenic differentiation, thereby alleviating osteoporosis (117–119). Phytol can prevent the accumulation of ROS by regulating the Nrf2/HO-1 pathway and restoring antioxidant enzymes, thereby improving osteoporosis (114).In addition, some TCMs, such as Qing’e pill, Bugu Shengsui Decoction, Zuogui Pills, etc., can prevent ferroptosis by regulating iron overload and lipid LOXs roxidation, thereby playing a role in preventing and treating osteoporosis (120–122). A summary of the various TCMs is found in **Table 2**.

**Table 2:** Summary of various traditional Chinese medicines.

TCMs	Regulatory pathway	Operation
Icariin	—	Increase ALP activity in osteoblasts Reduce oxidative damage caused by ferroptosis
Baicalein	SLC7A11/GPX4 Wnt/β-catenin	Inhibit the degradation of GPX4 Protect cell membrane from lipid peroxidation.
Quercetin	MAPK Wnt/β-catenin	Restoring GPX4 activity Reduce ROS and inflammatory response
Myricitrin	—	Reduce the production of reactive oxygen species Increase GSH activity Reducing the inflammatory response.
Benzoylaconitine	—	Increase the expression of GPX4 and β-catenin protein in osteoblasts
Duzhong-Xuduan	—	Inhibition of p53 protein expression level and reduce osteoblast apoptosis
Phytol	Nrf2/HO-1	Inhibit the accumulation of reactive oxygen species Restore antioxidant enzymes
Qing’e pill et al.	—	Prevent ferroptosis by regulating iron overload and lipid LOXs roxidation

## 5 Summarized and prospected

In recent years, ferroptosis has attracted wide attention as a new mechanism of cell death. Ferroptosis plays an important regulatory role in the occurrence and development of many diseases, especially liver disease and osteoporosis. Many studies have substantiated that iron overload and the accumulation of ROS constitute pivotal elements in the process of ferroptosis, thus establishing them as viable targets for the regulation of this distinct cell death mechanism. Iron chelator, melatonin and other medicines provide a new strategy for the prevention and treatment of osteoporosis by inhibiting iron overload, reducing ROS accumulation and interfering with multiple targets of ferroptosis. In addition, with the deepening of the research on the regulation of cell ferroptosis by traditional Chinese medicine preparations at home and abroad, the potential effects of traditional Chinese medicine components such as icariin, quercetin and myricitrin on osteoporosis have gradually emerged. However, the regulatory mechanisms and signaling pathways underlying ferroptosis in the context of osteoporosis remain incompletely elucidated. There remains a relative paucity of clinical studies investigating the application of traditional Chinese medicine preparations for the intervention of diseases associated with ferroptosis. The merits and demerits of both Chinese and Western medicinal preparations in the prevention and management of osteoporosis require further systematic summarization. In conclusion, future research is crucial to elucidate the precise mechanisms underlying ferroptosis in osteoporosis and to explore the regulatory mechanisms, as well as the advantages and disadvantages, of both Chinese and Western medicinal preparations in modulating ferroptosis. This will ultimately pave the way for novel insights and strategies in the prevention and treatment of osteoporosis.
